# A novel combination of serum microRNAs for the detection of early gastric cancer

**DOI:** 10.1007/s10120-021-01161-0

**Published:** 2021-03-20

**Authors:** Seiichiro Abe, Juntaro Matsuzaki, Kazuki Sudo, Ichiro Oda, Hitoshi Katai, Ken Kato, Satoko Takizawa, Hiromi Sakamoto, Fumitaka Takeshita, Shumpei Niida, Yutaka Saito, Takahiro Ochiya

**Affiliations:** 1 Endoscopy Division, National Cancer Center Hospital, Tokyo, Japan; 2 Division of Molecular and Cellular Medicine, National Cancer Center Research Institute, Tokyo, Japan; 3 Department of Breast and Medical Oncology, National Cancer Center Hospital, Tokyo, Japan; 4 Department of Gastric Surgery, National Cancer Center Hospital, Tokyo, Japan; 5 Department of Gastrointestinal Medical Oncology, National Cancer Center Hospital, Tokyo, Japan; 6 Toray Industries, Inc., Kanagawa, Japan; 7 Department of Biobank and Tissue Resources, Fundamental Innovative Oncology Core, National Cancer Center Research Institute, Tokyo, Japan; 8 Department of Translational Oncology, Fundamental Innovative Oncology Core, National Cancer Center Research Institute, Tokyo, Japan; 9 National Center for Geriatrics and Gerontology, Research Institute, Aichi, Japan; 10 Department of Molecular and Cellular Medicine, Tokyo Medical University, 6-7-1 Nishishinjuku, Shinjuku-ku, Tokyo, 160-0023 Japan

**Keywords:** microRNA, Microarray analysis, Early gastric cancer, Gastric cancer, Screening

## Abstract

**Background:**

The aim of this study was to identify serum miRNAs that discriminate early gastric cancer (EGC) samples from non-cancer controls using a large cohort.

**Methods:**

This retrospective case–control study included 1417 serum samples from patients with EGC (seen at the National Cancer Center Hospital in Tokyo between 2008 and 2012) and 1417 age- and gender-matched non-cancer controls. The samples were randomly assigned to discovery and validation sets and the miRNA expression profiles of whole serum samples were comprehensively evaluated using a highly sensitive DNA chip (3D-Gene^®^) designed to detect 2565 miRNA sequences. Diagnostic models were constructed using the levels of several miRNAs in the discovery set, and the diagnostic performance of the model was evaluated in the validation set.

**Results:**

The discovery set consisted of 708 samples from EGC patients and 709 samples from non-cancer controls, and the validation set consisted of 709 samples from EGC patients and 708 samples from non-cancer controls. The diagnostic EGC index was constructed using four miRNAs (miR-4257, miR-6785-5p, miR-187-5p, and miR-5739). In the discovery set, a receiver operating characteristic curve analysis of the EGC index revealed that the area under the curve (AUC) was 0.996 with a sensitivity of 0.983 and a specificity of 0.977. In the validation set, the AUC for the EGC index was 0.998 with a sensitivity of 0.996 and a specificity of 0.953.

**Conclusions:**

A novel combination of four serum miRNAs could be a useful non-invasive diagnostic biomarker to detect EGC with high accuracy. A multicenter prospective study is ongoing to confirm the present observations.

**Supplementary Information:**

The online version contains supplementary material available at 10.1007/s10120-021-01161-0.

## Introduction

Gastric cancer is the second most common malignancy and has a high mortality rate worldwide [1]. Although the incidence and mortality of gastric cancer have decreased gradually over the years, its burden has remained in East Asian countries. The prognosis of gastric cancer varies remarkably in relation to the stage of cancer, with 5-year survival rates of 90% and less than 5% in stages I and IV, respectively [2]. Thus, effective detection of EGC is essential to improve treatment outcomes and the quality of life for patients with gastric cancer.

The updated version of the Japanese Guidelines for Gastric Cancer Screening recommends radiographic and endoscopic screening as effective tools to detect EGC [3]; however, several groups have reported adverse events during gastric cancer screening, such as barium meal aspiration and intestinal obstruction during radiographic screening, nasal bleeding after transnasal endoscopy, and gastric mucosal laceration and post-biopsy bleeding after endoscopic screening [3, 4]. Although the overall complication rates are low (42.8/100,000 for radiographic screening and 87.4/100,000 for endoscopic screening), some adverse events can be serious, causing hospital admission or even death. Consequently, it is necessary to develop new screening methods for EGC with high sensitivities and specificities.

Several studies have investigated the role of circulating microRNAs (miRNAs), non-coding RNAs composed of 17–25 nucleotides, as diagnostic biomarkers of cancer [5, 6]. MiRNAs serve as a hub in gene regulatory networks by controlling numerous targets through RNA silencing and post-transcriptional regulation of gene expression [7]. Tissue-specific expression patterns of miRNAs are crucial for the precise regulation of cell differentiation and tissue development, alterations of which are involved in the pathogenesis of cancer [8, 9]. In addition, serum miRNAs can potentially be used as non-invasive biomarkers to detect cancer.

Our study group launched a national project in Japan entitled “Development and Diagnostic Technology for Detection of miRNA in Body Fluids”. This project includes a comprehensive characterization of the serum miRNA profiles of 13 types of human cancers, including EGC, in more than 10,000 patients using the same platform and technology. This aim of our current study was to develop a model to differentiate between EGC patients and non-cancer controls using the expression levels of serum miRNAs.

## Materials and methods

### Study population

**Fig. 1:**
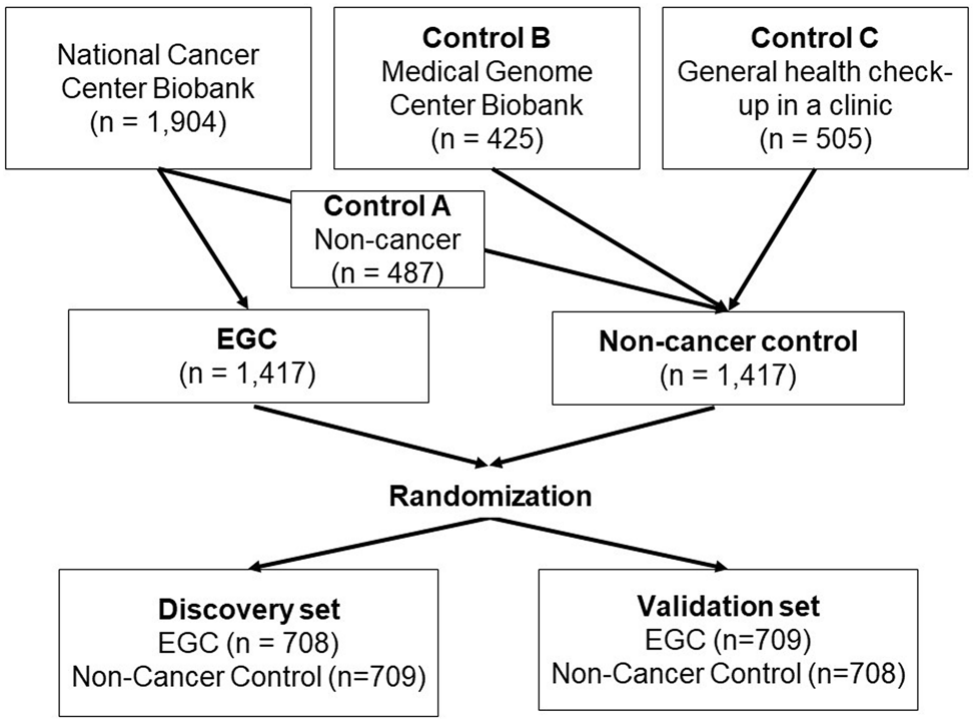
Flow chart of the development of the EGC index. The study included 1417 serum samples from EGC patients and 1417 non-cancer controls. The serum samples were randomly divided into the discovery and validation sets at a 1:1 ratio

This case–control study included 1417 serum samples from consecutive patients with initial EGC and without any other cancers at the National Cancer Center Hospital (Tokyo, Japan) between 2008 and 2012. All of the cancers were histologically proven and treated by either endoscopic resection or gastrectomy with lymph node dissection, according to the Japanese Gastric Cancer Treatment Guidelines [10]. Serum samples were obtained from all patients before treatment and were stored at − 20 °C in the National Cancer Center Biobank (Japan). Patients who had a history of any malignancy or who failed to give a serum sample or patient consent were excluded from the study. In addition, 1417 serum samples from age- and gender-matched non-cancer controls were randomly selected from our serum miRNA database. The controls consisted of 487 non-cancer patients with benign diseases of the prostate, bone and soft tissue, ovary, brain or breast recruited from the National Cancer Center Hospital between 2007 and 2016 (Control A), 425 individuals who visited the memory clinic from which serum samples were collected and stored at − 80 °C in the Biobank of the National Center for Geriatrics and Gerontology between 2012 and 2016 (Control B), and 505 healthy volunteers from which serum samples were collected during a general health check-up and stored at − 80 °C by Toray Industries and the Yokohama Minoru Clinic in 2015 (Control C). The serum samples from EGC patients and non-cancer controls were randomly divided into discovery and validation sets at a 1:1 ratio (Fig. 1).

The study was approved by the National Cancer Center Hospital Institutional Review Board (2015-266, 2016-249), the Research Ethics Committee of the Medical Corporation Shintokai Yokohama Minoru Clinic (6019-18-3772), and the Ethics and Conflict of Interest Committee of the National Center for Geriatrics and Gerontology (754). Written informed consent for the use of serum samples for research purposes was obtained from each participant.

### MiRNA extraction and microarray analysis

The standardized method of serum miRNA analysis has been published previously [11]. Briefly, **t**otal RNA was extracted from 300 µL of serum using 3D-Gene^®^ RNA extraction reagent (Toray Industries, Inc., Tokyo, Japan). A comprehensive miRNA microarray analysis was performed using a 3D-Gene^®^ Human miRNA Oligo Chip version 21 (Toray Industries, Inc.). The miRNA signal values were normalized to the ratio of the average signal value of three internal control miRNAs (miR-149-3p, miR-2861, and miR-4463) [12]. The validity of these control miRNAs was confirmed in the present dataset. To identify robust miRNAs, those with a normalized signal value exceeding 64 in more than 50% of the samples in each group were selected. Data sets analyzed in the study were submitted to the National Center for Biotechnology Information Gene Expression Omnibus database under accession number GSE164174.

### Statistical analysis

To establish a diagnostic model, the serum miRNA profiles of EGC patients and non-cancer controls in the discovery set were compared using Fisher’s linear discriminant analysis with a greedy algorithm, as reported previously [11]. The best combinations of miRNAs were selected based on their discrimination accuracy with leave-one-out cross-validation. Cut-off values were set at 0 based on the Youden index. The best discrimination model was selected by performing DeLong’s test on the discovery set. Finally, the discriminant model (named the EGC index) showing the highest statistically significant area under the receiver operating characteristic curve (AUC) was determined.

The diagnostic performance of the model was examined in the validation set. To confirm the robustness of the results, a subgroup analysis was performed for each non-cancer control group in the validation group. A subgroup analysis of EGC samples in the validation group was also performed for each pathological stage and histology classification. The clinicopathological information of EGC patients was collected from the hospital cancer registry. In patients with multiple EGCs, a main lesion was selected based on the depth of invasion and tumor size. The pathological stage was classified as IA, IB, or II, according to the guidelines of the International Union Against Cancer. The 7th edition clinical stage classification was used for patients who had a diagnosis of EGC in 2012, and the 6th edition was used for patients who had a diagnosis before 2012. The histology was classified into differentiated-type, undifferentiated type, or special-type, according to version 3 of the Japanese Classification of Gastric Cancer [13]. Continuous variables were compared using Student’s *t*-tests and categorical variables were compared using Pearson’s $\chi^{2}$tests.

Fisher’s linear discriminant analysis was performed using R version 3.6.3 (R Project for Statistical Computing) with compute.es package version 0.2-5, hash package version 2.2.6.1, MASS package version 7.3-51.5, mutoss package version 0.1-12, and pROC package version 1.16.2. Validation of internal control miRNAs was performed using the NormqPCR package in Bioconductor version 3.11. Principal component analyses and unsupervised clustering with Pearson's dissimilarity as a distance measure and Ward's method for linkage analysis were performed using Partek Genomics Suite 7.18.0723. All other statistical analyses were performed using IBM SPSS Statistics version 25 (IBM Japan). All *P*-values were reported as two-sided, and *P* < 0.05 was considered statistically significant.

## Results

### Characteristics of the control and EGC patients

This case–control study included 1417 serum samples from patients with EGC and 1417 age- and gender-matched controls. The samples were randomly divided into the discovery and validation sets (Fig. 1). The discovery set consisted of 708 samples from EGC patients and 709 samples from non-cancer controls, and the validation set consisted of 709 samples from EGC patients and 708 samples from non-cancer controls (Table 1). The majority of the EGC patients were pathologically stage IA. There were no significant differences in age, gender, stage, histologic-type, and tumor location between the discovery and validation sets.

**Table 1:** Clinicopathological characteristics of the study participants

	Discovery set (*n* = 1417)	Validation set (*n* = 1417)	*P value*
Early gastric cancer patients	(*n* = 708)	(*n* = 709)	
Age			
[mean (range)]	65.1 (22–89)	65.3 (20–90)	0.75^a^
Gender			
Male, %(*n*) Female, %(*n*)	72.2 (511) 27.8 (197)	69.1 (490) 30.9 (219)	0.21^b^
Stage			
IA, % (*n*) IB, % (*n*) II, % (*n*)	95.2 (674) 4.1 (29) 0.7 (5)	95.2 (675) 4.1 (29) 0.7 (5)	1.00^b^
Histology			
Differentiated-type, % (*n*) Undifferentiated-type, % (*n*) Special-types, % (*n*)	56.4 (399) 42.2 (299) 1.4 (10)	58.1 (412) 40.8 (289) 1.1 (8)	0.74^b^
Location			
Upper, % (*n*) Middle, % (*n*) Lower, % (*n*)	16.8 (119) 48.0 (340) 35.2 (249)	14.1 (100) 47.1 (334) 38.8 (275)	0.22^b^
Control patients without cancer	(*n* = 709)	(*n* = 708)	
Age			
[mean (range)]	65.5 (22–89)	64.5 (21–90)	0.07^a^
Gender			
Male, % (*n*) Female, % (*n*)	71.7 (508) 28.3 (201)	69.6 (493) 30.4 (215)	0.40^b^
Type of control			
National Cancer Center Biobank, % (*n*) Geriatrics and Gerontology,% (*n*) General health check-up in a clinic, % (*n*)	35.1 (249) 32.7 (232) 32.2 (228)	33.6 (238) 27.3 (193) 39.1 (277)	0.014^b^

^a^Student’s *t*-test

^b^Pearson’s $$\chi^{2}$$ test

**Table 2:** Best combination models of miRNAs in the discovery set

No. of miRNAs in the model	Model candidates	Sensitivity (95% C.I.)	Specificity (95% C.I.)	Accuracy (95% C.I.)	AUC (95% C.I.)	*P* value^a^ (Accuracy)	*P* value^b^ (AUC)
#1	(0.952637) × miR-6511b-5p-5.80077	0.934 (0.915–0.952)	0.872 (0.847–0.896)	0.903 (0.887–0.918)	0.958 (0.947–0.968)		
#2	(1.10492) × miR-6511b-5p + (− 0.924922) × miR-5739-0.3044826	0.951 (0.935–0.967)	0.946 (0.930­0.963)	0.948 (0.937–0.960)	0.983 (0.977–0.989)	3.0 × 10^–6^ (vs. #1)	3.1 × 10^–12^ (vs. #1)
#3	(0.636166) × miR-6511b-5p + (− 1.45364) × miR-5739 + (1.43993) × miR-4257-4.47133	0.972 (0.960–0.984)	0.948 (0.931–0.964)	0.960 (0.950–0.970)	0.990 (0.986–0.995)	0.15 (vs. #2)	0.0044 (vs. #2)
#4	(2.06054) × miR-4257 + (− 1.25451) × miR-6785-5p + (0.834875) × miR-187-5p + (− 1.07189) × miR-5739-4.4385	0.983 (0.974–0.993)	0.977 (0.966–0.988)	0.980 (0.973–0.987)	0.996 (0.993–0.999)	0.0014 (vs. #3)	0.0029 (vs. #3)
#5	(1.75411) × miR-4257 + (− .20966) × miR-6785-5p + (0.74851) × miR-187-5p + (− 1.16372) × miR-5739 + (0.960594) × miR-6075-9.69734	0.992 (0.985–0.998)	0.987 (0.979–0.996)	0.989 (0.984–0.995)	0.997 (0.994­1.000)	0.046 (vs. #4)	0.33 (vs. #4)

^a^Pearson’s $$\chi^{2}$$ test

^b^DeLong’s test

### Development of the EGC index

**Fig. 2:**
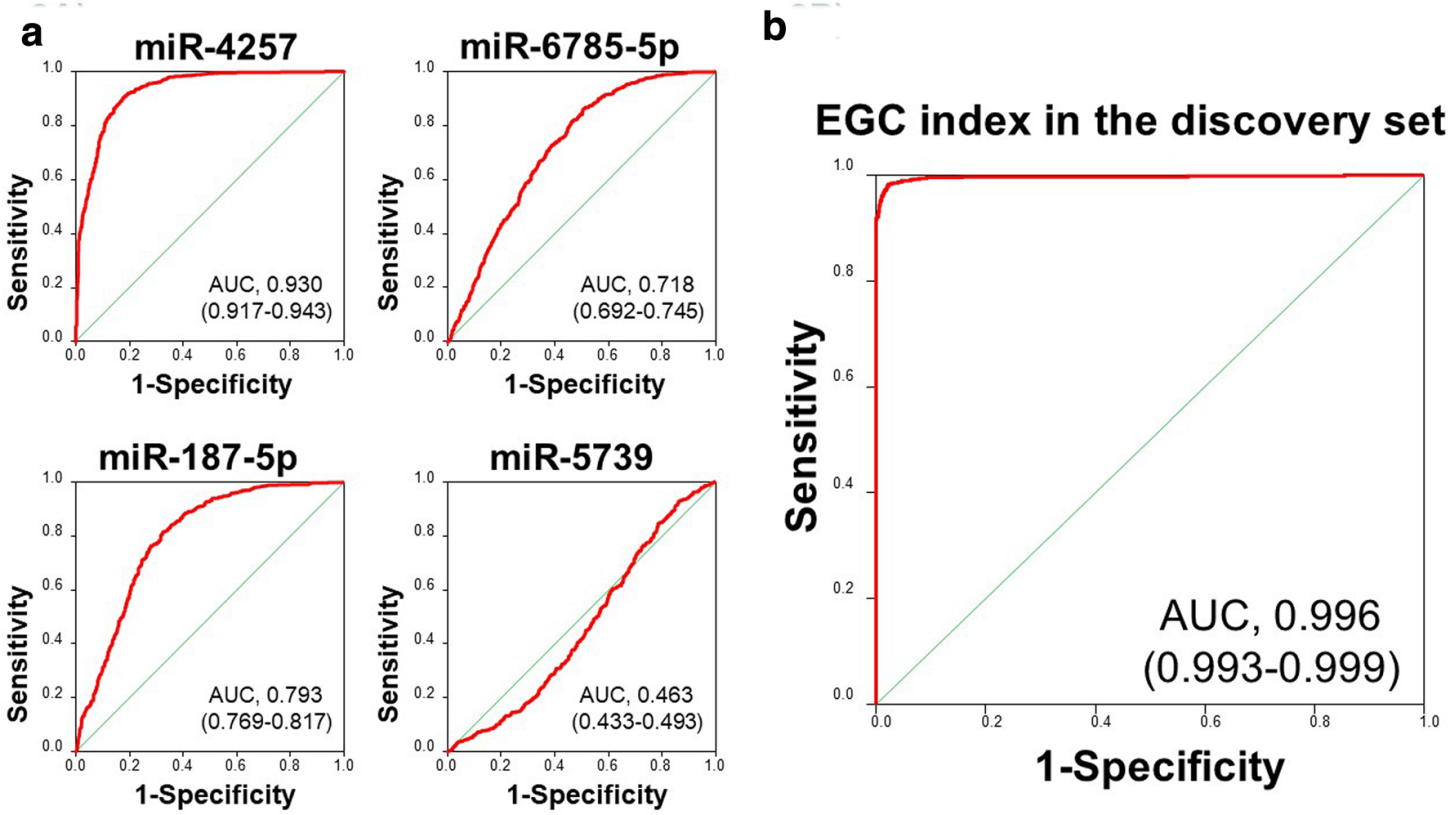
**a** The ability of each miRNA in the EGC index to distinguish between EGC and control samples in the discovery set. ROC analyses were used to determine the area under the curve (AUC) for each miRNA. The numbers in parentheses represent the 95% confidence intervals. **b**. ROC analysis of the EGC index in the discovery set. The numbers in parentheses represent the 95% confidence intervals of the area under the curve (AUC)

Among the 2565 miRNAs examined, 414 had a normalized signal value exceeding 64 in more than 50% of samples in each group and were selected for further analysis. The three control miRNAs (miR-149-3p, miR-2861, and miR-4463) were stably expressed in serum samples used in the present study (Supplementary Table 1). Table 2 lists the best combination models for discrimination of the EGC and control samples in the discovery set. We selected a model based on four miRNAs (miR-4257, miR-6785-5p, miR-187-5p, and miR-5739) as the EGC index because the AUC of this model was significantly higher than that of the three miRNA model (0.996 vs. 0.990, *P* = 0.0029), but was not significantly different to that of the five miRNA model (0.996 vs. 0.997, *P* = 0.33). The EGC index was calculated as follows: (2.06054) × miR-4257 + (− 1.25451) × miR-6785-5p + (0.834875) × miR-187-5p + (− 1.07189) × miR-5739–4.4385. This index achieved a sensitivity of 0.983 and a specificity of 0.977. Figure 2a shows the ability of each of the four miRNAs in the EC index to distinguish between the EGC and control samples in the discovery set. Receiver operating characteristic curve (ROC) analyses showed that the AUC for each miRNA varied from 0.463 to 0.930, indicating a range of discriminative abilities. The EGC index including all four of these miRNAs demonstrated significantly better diagnostic accuracy than each miRNA alone (AUC 0.996; 95% C.I. 0.993–0.999) (Fig. 2).

### Validation of the EGC index

**Fig. 3:**
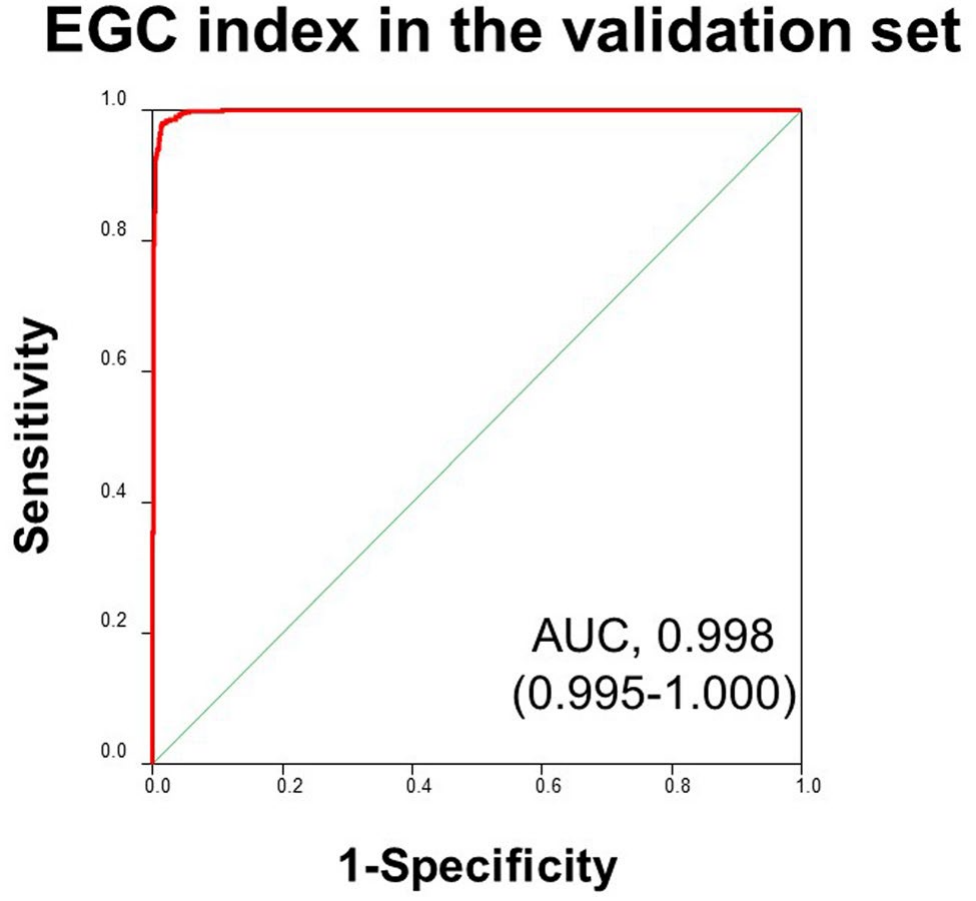
ROC analysis of the EGC index in the validation set. The numbers in parentheses represent the 95% confidence intervals of the area under the curve (AUC)

**Fig. 4:**
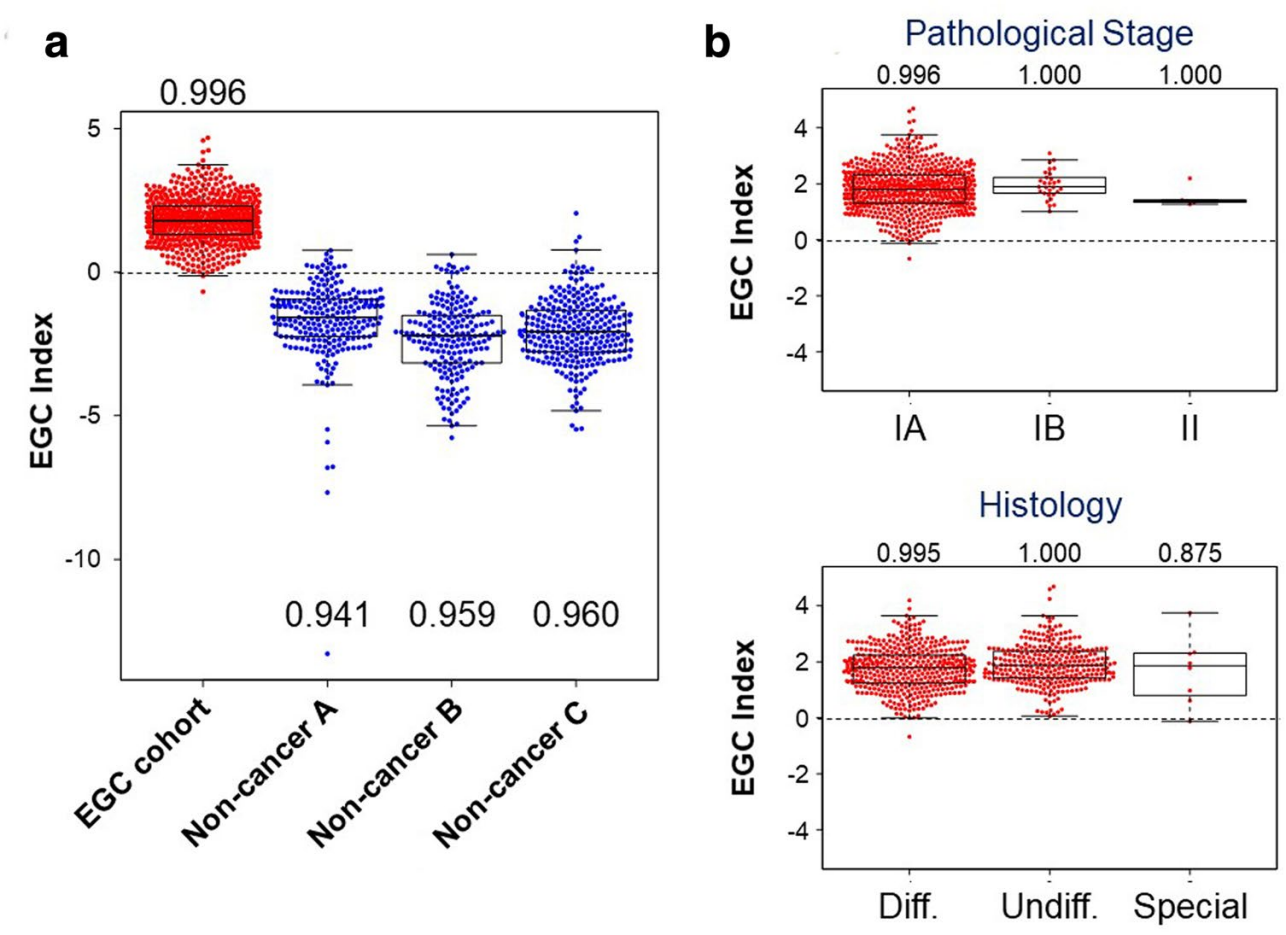
**a** Bee swarm plots of the EC index in EGC samples and each non-cancer control group. The numbers indicate the specificity for each control group. **b **Bee swarm plots of the EGC index according to pathological stage (IA, IB, or II) and histology (differentiated-type, undifferentiated-type, and special-type). The numbers indicate the specificity for each group

**Fig. 5:**
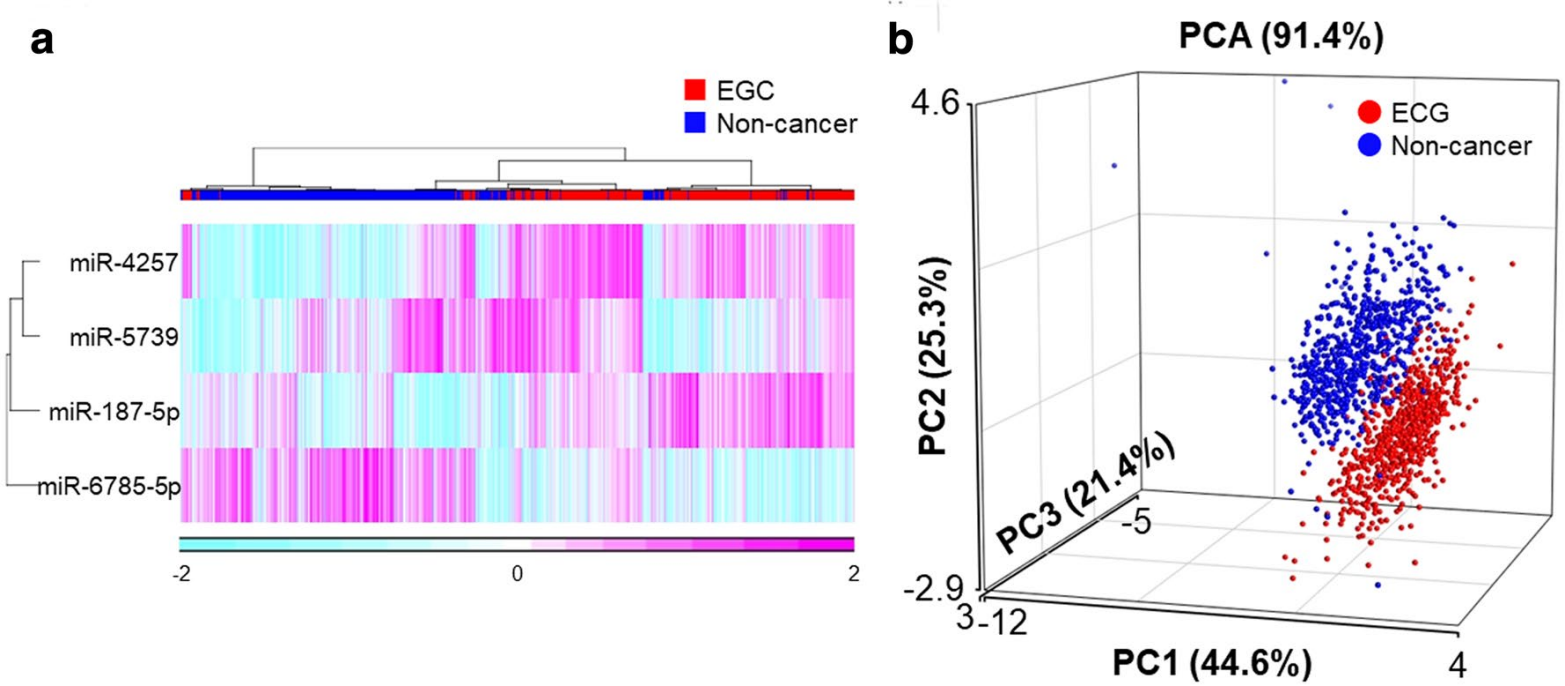
**a **Unsupervised hierarchical clustering analysis of the four miRNAs in the EGC index. The EGC and non-cancer control samples in the validation set were plotted. The levels of miRNAs were standardized by considering the mean as 0 and the standard deviation as 1 in all features. **b** Principal component analysis using the levels of the four miRNAs in the EGC index. The axes show the first three principal components, which account for 91.4% of the variance. The percentages of explained variance for each principal component are indicated

In the validation set, the AUC for the EGC index was 0.998 (95% C.I. 0.995–1.000), with a sensitivity of 0.996 (95% C.I. 0.991–1.000) and a specificity of 0.953 (95% C.I. 0.938–0.969) (Fig. 3). Figure 4a shows bee swarm plots of the EGC index in the EGC cohort and each non-cancer control group. The specificity ranged from 0.941 to 0.960 in each control group. The sensitivity by pathological stage was 0.996 for stage IA, 1.000 for stage IB, and 1.000 for stage II. As for the histological type, the sensitivity was 0.995 for differentiated-type adenocarcinoma, 1.000 for undifferentiated-type, and 0.875 for special-type (Fig. 4b). Unsupervised hierarchical clustering and a principal component analysis showed that the four miRNAs in the EGC index effectively differentiated EGC samples from non-cancer controls (Fig. 5).

## Discussion

In the current study, we developed an EGC index to differentiate EGC from non-cancer controls based on the serum levels of four miRNAs (miR-4257, miR-6785-5p, miR-187-5p, and miR 5739). In the validation set, the EGC index demonstrated a sensitivity of 0.996 and a specificity of 0.953, with an AUC of 0.998. The sensitivities of the EGC index did not differ significantly among the clinical stages of EGC or between the three sets of non-cancer control samples.

The updated version of the Japanese Guidelines for Gastric Cancer Screening recommends performing an upper gastrointestinal series and gastroscopy for population-based and opportunistic gastric cancer screenings [3]. Some large-scale cohort studies have reported that both of these screening modalities contribute to the reduction of gastric cancer mortality [14–16], although there were several inconsistent results among the studies. Hamashima et al. reported that the sensitivities of radiographic and endoscopic screening methods for EGC detection were 0.893 (95% C.I. 0.718–0.977) and 0.955 (95% C.I. 0.875–0.991), respectively, with specificities of 0.856 (95% C.I. 0.846–0.865) and 0.851 (95% C.I. 0.843–0.859), respectively [17]. In the study by Hamashima et al., the false-negative rates in the first round were 10.7% and 4.5% for radiographic and endoscopic screening, respectively, and the false-positive rates in the first round were 14.4 and 14.9%, respectively [17]. Notably, EGC is easily missed during screening, even when it is performed by qualified endoscopists [18–20]. Despite its inability to visualize the target, the EGC index developed here could be used as an alternative non-invasive screening modality for the detection of EGC. In the validation set of our study, the AUC for the EGC index was 0.998 (95% C.I. 0.995–1.000), with a sensitivity of 0.996 (95% C.I. 0.991–1.000) and a specificity of 0.953 (95% C.I. 0.938–0.969). When we consider the prevalence of gastric cancer as 0.742% in a screening population in Japan according to Hamashima et al. [17], the positive predictive value (PPV) and negative predictive value were 0.138 (95% C.I. 0.103–0.182) and 1.00 (95% C.I. 0.999–1.00), respectively. The PPV of the EGC index was higher than those of the endoscopic screening (0.055) and the radiographic screening (0.031) [17].

*Helicobacter pylori* infection is a well-known risk factor for gastric cancer, and intestinal metaplasia is one of the most common pre-cancerous lesions that may lead to the disease [21]. A combination of detecting serum antibodies against *H. pylori* and measuring the level of serum pepsinogens is a method of screening for EGC [22, 23]. Although this combination method is non-invasive, it is designed for the risk stratification of gastric cancer rather than its detection. The Japanese Guidelines for Gastric Cancer Screening does not recommend the combination method for population-based screening because there is insufficient evidence that it reduces the mortality of gastric cancer [3]. The EGC index could be utilized not as a risk stratification method, but as a sensitive screening modality with high specificity. The use of non-invasive diagnostic biomarkers could contribute to the detection of EGC detection and hence improve medical management of the disease.

A variety of serum or plasma miRNAs are frequently upregulated or downregulated in gastric cancer [24]. Although several groups have investigated the use of serum miRNAs to detect gastric cancer and predict the recurrence and prognosis of the disease [25–27]. To our knowledge, our current study is the largest cohort analysis of the use of serum miRNAs to detect EGC with the highest sensitivity and specificity reported to date. So et al. recently developed a clinical assay for the detection of gastric cancer based on a 12-miRNA Biomarker panel with AUCs of 0.93 and 0.92 in the discovery and verification cohorts, respectively. Although the sample size in the training set was smaller and AUC was lower in their study than in our study, the 12-miR assay was validated and cost-effectiveness was analyzed in a large prospective validation cohort consisting of 5282 participants [27].

The EGC index developed here includes four miRNAs: miR-4257, miR-6875-5p, miR-187-5p, and miR-5739. The serum levels of miR-4257 and miR-187-5p were higher in the EGC samples than in the non-cancer control samples, whereas the level of miR-6785-5p was lower in the EGC samples than in the control samples. Although the levels of miR-5739 were comparable in the EGC and control samples (AUC 0.463), AUC of the EGC index was higher when this miRNA was included than when it was excluded (Supplementary Table 2). Although the roles of these miRNAs in carcinogenesis remain unclear, some previous reports support the results of our study. Notably, miR-187-5p has already been described as a serum biomarker for the early detection of gastric cancer [28]. In a study by Wang et al., the expression level of miR-187-5p was significantly lower in diffuse-type gastric cancer tissue than in normal gastric tissue [29], suggesting that the damaged gastric tissue surrounding the cancer site could release miR-187-5p. Notably, exosomes derived from normal gastric epithelial cells function to inhibit the progression of gastric cancer [30, 31]. miR-187-5p has a tumor-suppressive effect in non-small-cell lung cancer [32]; therefore, the active release of miR-187-5p from the tumor microenvironment might play a role in suppressing tumor growth. It was difficult to explain why the miR-187-5p level was higher in the EGC samples than in the non-cancer control samples in this study. Furthermore, Shuai et al. reported that miR-6785-5p suppresses tumor growth by targeting BCL2 [33] and demonstrated that the long non-coding RNA MNX1-AS1, which is highly expressed in gastric cancer tissue, can suppress the function of miR-6875-5p in gastric cancer cells. This mechanism could possibly explain why the serum levels of miR-6875-5p were lower in the EGC samples than in the control samples in the present study. We were unable to find any publications related to the roles of miR-4257 and mir-5739 in gastric cancer; therefore, further studies of their functions and methods of regulation are warranted.

It is important to analyze the diagnostic performance of the EGC index in advanced gastric cancer and other malignancies for further discrimination of EGC. Exploratory data were analyzed in ten patients with serum samples of Stage III locally advanced gastric cancer, 50 esophageal squamous cell carcinomas (ESCC), and 50 colorectal cancers (CRC). The AUCs of the EGC index in Stage III gastric cancer, ESCC, and CRC were 1.00, 0.640, and 0.440, respectively (Supplementary Fig. 1A, B). Further discrimination models should be established in a large-scale cohort study.

Our current study has several limitations. First, the study was a retrospective analysis using archival samples, and an external validation cohort for patients with EGC was not available. Although the reproducibility of 3D-Gene^®^ in the diagnostic index of prostate cancer was reported previously by our study group [11], further investigations are warranted to confirm the reproducibility of the EGC index. Second, variations in sample collection and storage may have influenced the EGC index because samples were collected from three different institutions. To confirm that the EGC index can discriminate not only external controls but also internal controls, our control set included serum samples from patients with benign diseases from the National Cancer Center Hospital. Third, although we performed a comprehensive analysis of miRNAs using age- and gender-matched EGC and non-cancer control samples, we were not able to evaluate other well-known risk factors of gastric cancer, such as *H. pylori* infection, atrophic gastritis, and smoking, which could have influenced the levels of circulating miRNAs [22, 34], because the data of these risk factors were unavailable owing to the retrospective nature of data collection in this study. To overcome these limitations, we have recently conducted a prospective confirmatory study using serum samples from multiple institutions.

In conclusion, the novel combination of serum miRNAs comprising miR-4257, miR-6785-5p, miR-187-5p, and miR 5739 could be a useful diagnostic biomarker to detect EGC with high accuracy.

## Supplementary Information

Below is the link to the electronic supplementary material.

Supplementary file1 (PDF 1334 KB)

Supplementary file2 (DOCX 17 KB)

Supplementary file3 (JPG 1232 KB)

## Abbreviations

AUC: Area under the curve
EGC: Early gastric cancer
miRNA: microRNA
ROC: Receiver operating characteristic curve
