# Supplementary material for: A novel combination of serum microRNAs for the detection of early gastric cancer
Source: Gastric Cancer. 2021 Mar 20;24(4):835–43. doi: 10.1007/s10120-021-01161-0 (PMC8205917; doi:10.1007/s10120-021-01161-0)
Supplement: Supplementary file 2 — Supplementary file2 (DOCX 17 KB) [file 10120_2021_1161_MOESM2_ESM.docx]

**Supplementary Table 1: Ranking of stably-expressed housekeeping micro RNAs**

|  | National Cancer  Center Biobank | Control B | Control C |
| --- | --- | --- | --- |
| miR-149-3p # | 1 | 33 | 37 |
| miR-4763-3p | 2 | 61 | 59 |
| miR-1268b | 3 | 36 | 13 |
| miR-6085 | 4 | 54 | 56 |
| miR-6798-5p | 5 | 56 | 43 |
| miR-6879-5p | 6 | 77 | 83 |
| miR-4530 | 7 | 90 | 84 |
| miR-6791-5p | 8 | 57 | 50 |
| miR-7108-5p | 9 | 38 | 45 |
| miR-1343-5p | 10 | 42 | 41 |
| miR-1268a | 11 | 44 | 14 |
| miR-6781-5p | 12 | 30 | 19 |
| miR-6816-5p | 13 | 18 | 23 |
| miR-6803-5p | 14 | 12 | 9 |
| miR-4442 | 15 | 68 | 66 |
| miR-6768-5p | 16 | 72 | 75 |
| miR-3197 | 17 | 67 | 62 |
| miR-5001-5p | 18 | 71 | 78 |
| miR-4665-5p | 19 | 69 | 76 |
| miR-4505 | 20 | 62 | 74 |
| miR-6743-5p | 21 | 64 | 68 |
| miR-4294 | 22 | 82 | 91 |
| miR-6780b-5p | 23 | 83 | 82 |
| miR-6765-5p | 24 | 29 | 27 |
| miR-6724-5p | 25 | 49 | 63 |
| miR-4463 # | 26 | 40 | 48 |
| miR-1227-5p | 27 | 26 | 32 |
| miR-6752-5p | 28 | 27 | 15 |
| miR-4467 | 29 | 84 | 88 |
| miR-3656 | 30 | 17 | 16 |
| miR-1908-5p | 31 | 35 | 36 |
| miR-4492 | 32 | 24 | 47 |
| miR-6126 | 33 | 79 | 77 |
| miR-6805-5p | 34 | 34 | 40 |
| miR-4484 | 35 | 73 | 61 |
| miR-6786-5p | 36 | 8 | 2 |
| miR-6125 | 37 | 11 | 12 |
| miR-6749-5p | 38 | 39 | 51 |
| miR-8072 | 39 | 14 | 42 |
| miR-6789-5p | 40 | 25 | 31 |
| miR-6850-5p | 41 | 1 | 18 |
| miR-3196 | 42 | 19 | 44 |
| miR-2861 # | 43 | 21 | 22 |
| miR-1228-5p | 44 | 53 | 38 |
| miR-1237-5p | 45 | 15 | 46 |
| miR-328-5p | 46 | 28 | 33 |
| miR-4651 | 47 | 31 | 39 |
| miR-3940-5p | 48 | 20 | 21 |
| miR-6729-5p | 49 | 6 | 7 |
| miR-4466 | 50 | 7 | 17 |

**Supplementary Table 2: The combination models of miRNAs with and without miR-5739 in the discovery set**

|  | **Model candidates** | **Sensitivity** | **Specificity** | **Accuracy** | **AUC** | ***P-value ^a^*** |
| --- | --- | --- | --- | --- | --- | --- |
|  |  | **(95% C.I.)** | **(95% C.I.)** | **(95% C.I.)** | **(95% C.I.)** |  |
| **EGC index** | (2.06054)×miR-4257+(-1.25451)×miR-6785-5p+(0.834875)×miR-187-5p+(-1.07189)×miR-5739-4.4385 | 0.983 | 0.977 | 0.98 | 0.996 |  |
|  |  | (0.974­0.993) | (0.966­0.988) | (0.973­0.987) | (0.993­0.999) |  |
| **EGC index without miR-5739** | (1.61158)×miR-4257+(-1.46189)×miR-6785-5p+(0.781931)×miR-187-5p-6.55283 | 0.979 | 0.966 | 0.973 | 0.993 | 0.00091 |
|  |  | (0.968­0.989) | (0.953­0.980) | (0.964­0.981) | (0.988­0.997) | (vs. EGC index) |
