# Supplementary figures and images for: A novel combination of serum microRNAs for the detection of early gastric cancer
Source: Gastric Cancer. 2021 Mar 20;24(4):835–43. doi: 10.1007/s10120-021-01161-0 (PMC8205917; doi:10.1007/s10120-021-01161-0)

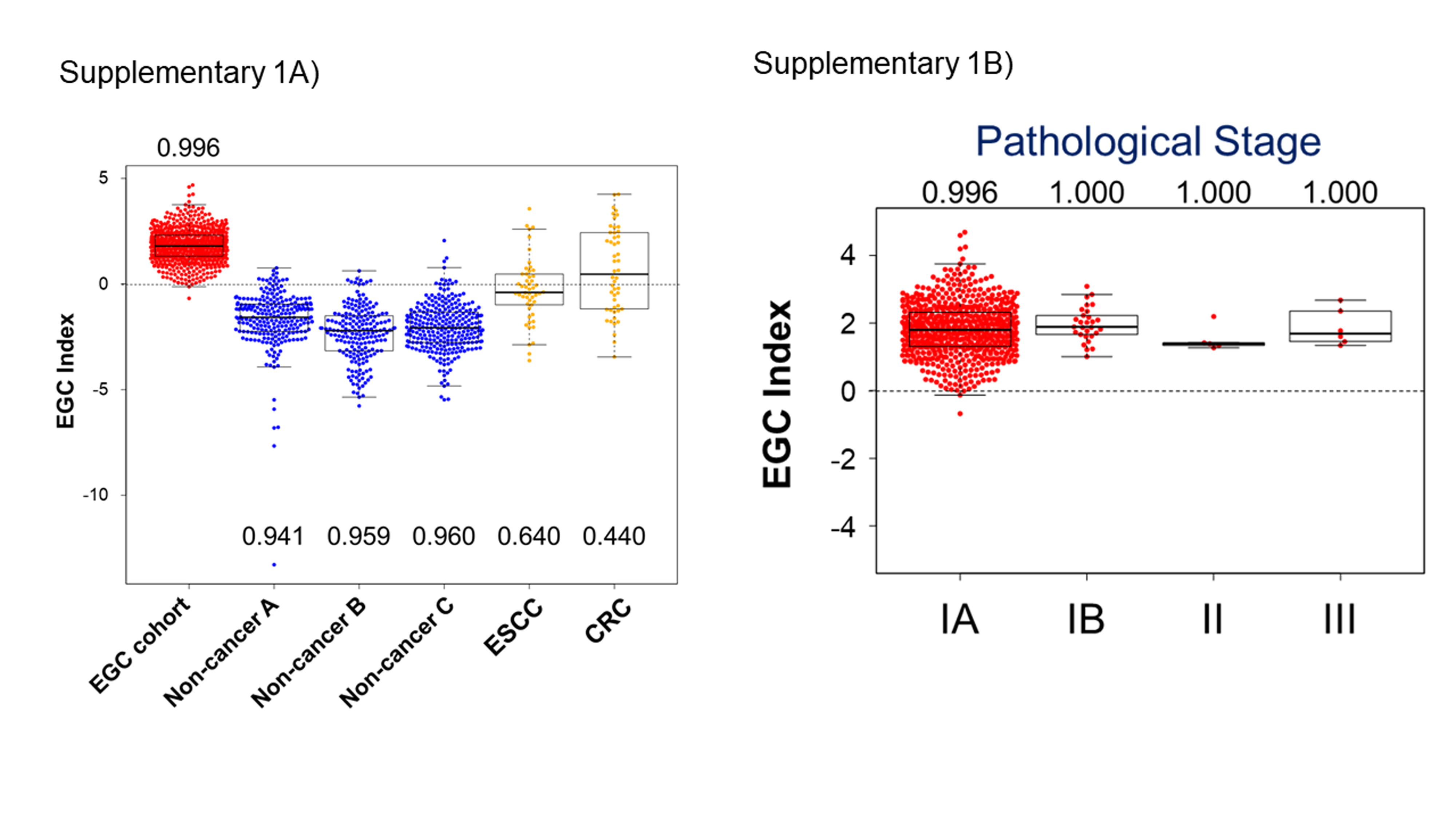

Supplement: Supplementary file 3 — Supplementary file3 (JPG 1232 KB) [file 10120_2021_1161_MOESM3_ESM.jpg]
